# Epidemiological cluster identification using multiple data sources: an approach using logistic regression

**DOI:** 10.1099/mgen.0.000929

**Published:** 2023-03-03

**Authors:** Kurnia Susvitasari, Paul F. Tupper, Irving Cancino-Muños, Mariana G. Lòpez, Iñaki Comas, Caroline Colijn

**Affiliations:** ^1^​ Department of Mathematics, Simon Fraser University, Burnaby, Canada; ^2^​ I2SysBio, University of Valencia-CSIC, Valencia, Spain; ^3^​ FISABIO Public Health, Valencia, Spain; ^4^​ Tuberculosis Genomics Unit, Instituto de Biomedicina de Valencia (IBV-CSIC), Valencia, Spain; ^5^​ Ciber en Epidemiología y Salud Pública (CIBERESP), Madrid, Spain

**Keywords:** genomic clustering, TB cases

## Abstract

In the management of infectious disease outbreaks, grouping cases into clusters and understanding their underlying epidemiology are fundamental tasks. In genomic epidemiology, clusters are typically identified either using pathogen sequences alone or with sequences in combination with epidemiological data such as location and time of collection. However, it may not be feasible to culture and sequence all pathogen isolates, so sequence data may not be available for all cases. This presents challenges for identifying clusters and understanding epidemiology, because these cases may be important for transmission. Demographic, clinical and location data are likely to be available for unsequenced cases, and comprise partial information about their clustering. Here, we use statistical modelling to assign unsequenced cases to clusters already identified by genomic methods, assuming that a more direct method of linking individuals, such as contact tracing, is not available. We build our model on pairwise similarity between cases to predict whether cases cluster together, in contrast to using individual case data to predict the cases’ clusters. We then develop methods that allow us to determine whether a pair of unsequenced cases are likely to cluster together, to group them into their most probable clusters, to identify which are most likely to be members of a specific (known) cluster, and to estimate the true size of a known cluster given a set of unsequenced cases. We apply our method to tuberculosis data from Valencia, Spain. Among other applications, we find that clustering can be predicted successfully using spatial distance between cases and whether nationality is the same. We can identify the correct cluster for an unsequenced case, among 38 possible clusters, with an accuracy of approximately 35 %, higher than both direct multinomial regression (17 %) and random selection (< 5 %).

## Data Summary

All sequencing data in this study have been deposited to the European Nucleotide Archive (ENA) under accession codes PRJEB29604 and PRJEB38719, and the epidemiological data on which the analyses are performed is available in Cancino-Muñoz *et al*. [1]. The method introduced in the study is made available as an R package called lr2cluster. All scripts to reproduce the figures and tables in the study are available in the GitHub repository under the project’s name Genomic-Clustering.

**Impact Statement**

In managing infectious diseases with genomic epidemiology, grouping cases into clusters of linked individuals is a fundamental task. With whole-genome sequencing of all cases, genomic data can be used to identify clusters. But often sequencing data are incomplete. This poses challenges for public health analyses that rely on clusters. Meanwhile, other sources of data are likely to be available: time and location of sample collection, demographic data such as gender and nationality, or clinical data including the presence of other diseases. We show how to utilize these other data types in order to assign cases to clusters when high-quality genomics or contact tracing data are unavailable. Our key method is to train a logistic regression model to predict whether two cases are in the same cluster given the dissimilarity between their available data. We explore various applications of our method with tuberculosis data from Valencia, Spain.

## Introduction

In the context of infectious diseases, clusters are groups of cases that are likely to be linked, either through direct infection or by short transmission chains [2]. Assigning cases to clusters is an important aspect of genomic epidemiology. Knowing how cases are clustered can be an important resource for public health investigators [3–5]; if most cases in a region are in one large cluster, this suggests substantial recent transmission, whereas many small and very distinct clusters suggest multiple introductions followed by less local transmission. Cluster assignment can be done with epidemiological data (exposure setting, time of sample collection, contact tracing), with these data combined with molecular typing data, or primarily with molecular data, including in the form of whole-genome sequencing (WGS). WGS has high resolution compared to conventional molecular typing, and can help to identify closely related cases.

Where genetic data are available, sequence or type similarity can be used to define clusters, to detect probable transmission events, and to exclude the possibility of direct transmission [6–8]. Stimson *et al*. [9] and McCloskey *et al*. [10] have developed methods to identify clusters using WGS data, allowing high-resolution definition of clusters. However, WGS data are often not available for all cases. This motivates incorporating other sources of data: several approaches have been developed in this direction, incorporating temporal data [11–17], spatial data [18–20], or by combining several data streams [21–23]. For chronic infections such as tuberculosis, however, in which individuals may carry an infection for a long period of time, the time of diagnosis may not be particularly informative.

Assuming that data to directly determine links among cases – such as contact tracing data – are not available, we introduce a new framework for using multiple available data sources to place unassigned cases into clusters. Our approach is to view cluster assignment as a classification problem and apply logistic regression. For each case, we have individual data which may include demographic, clinical, or location data. Then for each pair of cases, we define pairwise variables describing the dissimilarity between two cases, using their individual data. We then train and test logistic regression models to predict whether two cases are in the same genomic cluster using their pairwise data. For example, one piece of individual data might be geographical location. For each pair, we can then define the pairwise variables, in this case, the spatial distance between the two cases. We train our model using cases where we know whether the two cases are in the same cluster. In practice, this may be when we have genetic or epidemiological data to define the clusters. In addition to these applications, logistic regression potentially allows us to identify which variables are important for determining whether two cases are in the same cluster.

We apply our method to tuberculosis (TB) clusters and cases from Valencia, Spain, over a period between 2014 and 2016, using clinical and demographic data, together with clusters defined using WGS data. We develop several applications oriented towards the situation where WGS is available only for a subset of isolates. For example, we examine pairs of unsequenced cases and use the model to predict whether they cluster together (potentially allowing the identification of new clusters), allocate unsequenced cases to their probable clusters, and probe which cases, of a collection of unsequenced cases, are likely to be members of a specific cluster of interest. Finally, we estimate the true sizes of clusters (accounting for unsequenced cases that are not yet allocated to clusters). We then compare our approach with cluster prediction using multinomial logistic regression on individual data, and with random cluster assignment as a benchmark.

## Methods

### Pairwise data and the logistic regression model

For each detected case of the infectious disease in question, we have a vector of data that may contain demographic data, contact/social network data, or relevant clinical information, all of which we refer to as individual data. Then for each pair of cases, we compute a vector, each component of which gives a measure of the dissimilarity between the two cases’ individual data. We refer to this as the pairwise data. Our method trains a logistic regression model to infer whether two cases are in the same cluster using pairwise data.

Specifically, let ***x***_*i*_ = (*x*_i1_,*x*_i2_, …, *x*_ip_) be the individual data of case i . We define the pairwise variables of case-pair (*i*, *j*), denoted by ***z***_*(ij)*_ = (*z*_(*ij*),2_, …, *z*_(*ij*),*m*_). If *x*_ik_ is a continuous variable we define an element of the pairwise vector to be

(1) $$\begin{array}{r} z_{(ij),k}=d(x_{ik},x_{jk}),\;k=1,2,\ldots ,m. \end{array}$$

where *d*(·) is a measure of dissimilarity. For example, if *x*_*ik*_ is location, we define *d*(*x*_*ik*_, *x_jk_*) to be the distance between the two locations. In the case where the individual variable *x*_*ik*_ is binary, say taking values in {0, 1}, we define two pairwise variables to define *x*_*ik*_. For example, suppose *x*_*ik*_ = 0 represents the gender of case i to be female, and *x*_*ik*_ = 1 to be male. Then, we will have two binary variables: z_(*ij*),k=0_ and z_(*ij*),k=1_ to define the gender similarity between case *i* and case *j* as follows

**Table:** 

*x*_ik_	*x*_jk_	Description	Pairwise representation
0	0	*i* and *j* are female	*z*_(*ij*),k=0_ = 1, *z*_(*ij*),k=1_ = 0
1	1	*i* and *j* are male	*z*_*(ij*),k=0_ = 0, *z*_(*ij*),k=1_ = 1
0	1	*i* and *j* have different gender	*z*_(*ij*),k=0_ = 0, *z*_(*ij*),k=1_ = 0
1	0		

Without losing generality, if, for example, *x*_*ik*_ has *h*>2 categorical values, we could define *h* pairwise variables in a similar way as above, though we do not consider this possibility further here (see “Tuberculosis data in Valencia”). For case pairs (*i*, *j*) where we know the cluster assignment of both cases, we also define a binary response variable

(2) $$\begin{array}{r} y_{\left(ij\right)}=\left\{ \begin{array}{l} 1,\;i\text{ and }j\text{ in the same cluster}\\ 0,\;\text{otherwise}. \end{array}\right. \end{array}$$

We train a logistic regression model that uses the pairwise data of a pair of cases to estimate the probability that two cases are in the same cluster. For *m*-dimensional pairwise data ***z***_(*ij*)_ and cluster assignment data *y*_(*ij*)_, the model is

(3) $$\begin{array}{r} \pi_{\left(ij\right)}\equiv P(y_{\left(ij\right)}=1|{\text{z}}_{\left(ij\right)})=\frac{\exp(\beta_{0}+\sum_{k=1}^{m}\beta_{k}z_{(ij)k})}{1+\exp(\beta_{0}+\sum_{k=1}^{m}\beta_{k}z_{(ij)k})}. \end{array}$$

The maximum likelihood estimate (MLE) of the parameters *β* is obtained by maximizing the following log-likelihood function

(4) $$\begin{array}{r} \ell (\beta )=\sum_{\left(ij\right)}y_{\left(ij\right)}\log\left(\pi_{\left(ij\right)}\right)+(1-y_{\left(ij\right)})\log(1-\pi_{\left(ij\right)}). \end{array}$$

Furthermore, performing a Wald test [24, p. 152], which is used to test the relationship between the response variable and the predictor variables, on each estimated *β* will allow us to examine which variables give strong signal in determining whether two cases are in the same cluster.

We compare our method to a multinomial logistic regression in which we use individual data to directly predict the cluster for each case and random cluster assignment in which we randomly assign cases to clusters with probability proportional to their cluster sizes. Since our method is implemented on pairwise data, we will refer to our method as pairwise logistic regression to distinguish it from multinomial logistic regression on individual data.

### Applications of pairwise logistic regression

There are four main applications of the model, answering different questions related to unsequenced cases.

#### Do two new cases belong to the same cluster?

A direct application of the model (3) is to predict whether a pair of new cases cluster together. They may be from a new cluster entirely, not yet known from the set of cases that have been sequenced; see “Which cluster is an unsequenced case most likely to belong to?”.

For any pair of new cases, say *i* and *j*, we compute the pairwise data **z**_(*ij*)_ and, using the model (3), obtain a probability π_(*ij*)_. To make an actual decision of whether we think they are in the same cluster or not, we need to select a threshold or discrimination value π_0_. If π_(*ij*)_ > π_0_, we consider *i* and *j* to be in the same cluster, whereas if π_(*ij*)_ ≤ π_0_, we consider them to be in different clusters.

As in any binary classification problem where a threshold must be selected, the optimal threshold depends on how the user values the cost of false positive errors (saying two cases are in the same cluster when they are not) versus false negative errors (saying two cases are not in the same cluster when they are). Selecting π_0_ to maximize Youden’s J statistic [25] is often used. An extension of this method is to include the costs of different types of incorrect classifications, yielding the generalized Youden’s index (GYI) [26, 27]. We will use the latter method to find π_0_ by maximizing the following

(5) $$\text{GYI}(\pi_{0})=\text{Se}(\pi_{0})+\frac{1-p}{cp}\text{Sp}(\pi_{0})-1$$

where Se(π_0_) and Sp(π_0_) are the sensitivity and specificity evaluated when the threshold is π_0_, *p* is the prevalence (estimated using the proportion of the number of pairs in the same cluster over the total pairs in the training set), and *c* denotes the relative loss (cost) of false negative classification as compared with false positive classification. To test the performance of our model, we train the model on the training set, including setting the threshold π_0_, and we predict the probability of being in the same cluster for cases in the test set. The accuracy, as well as the sensitivity and specificity evaluated at the optimum threshold π_0_, are used to assess our model’s performance.

#### Which cluster is an unsequenced case most likely to belong to?

**Fig. 1.**
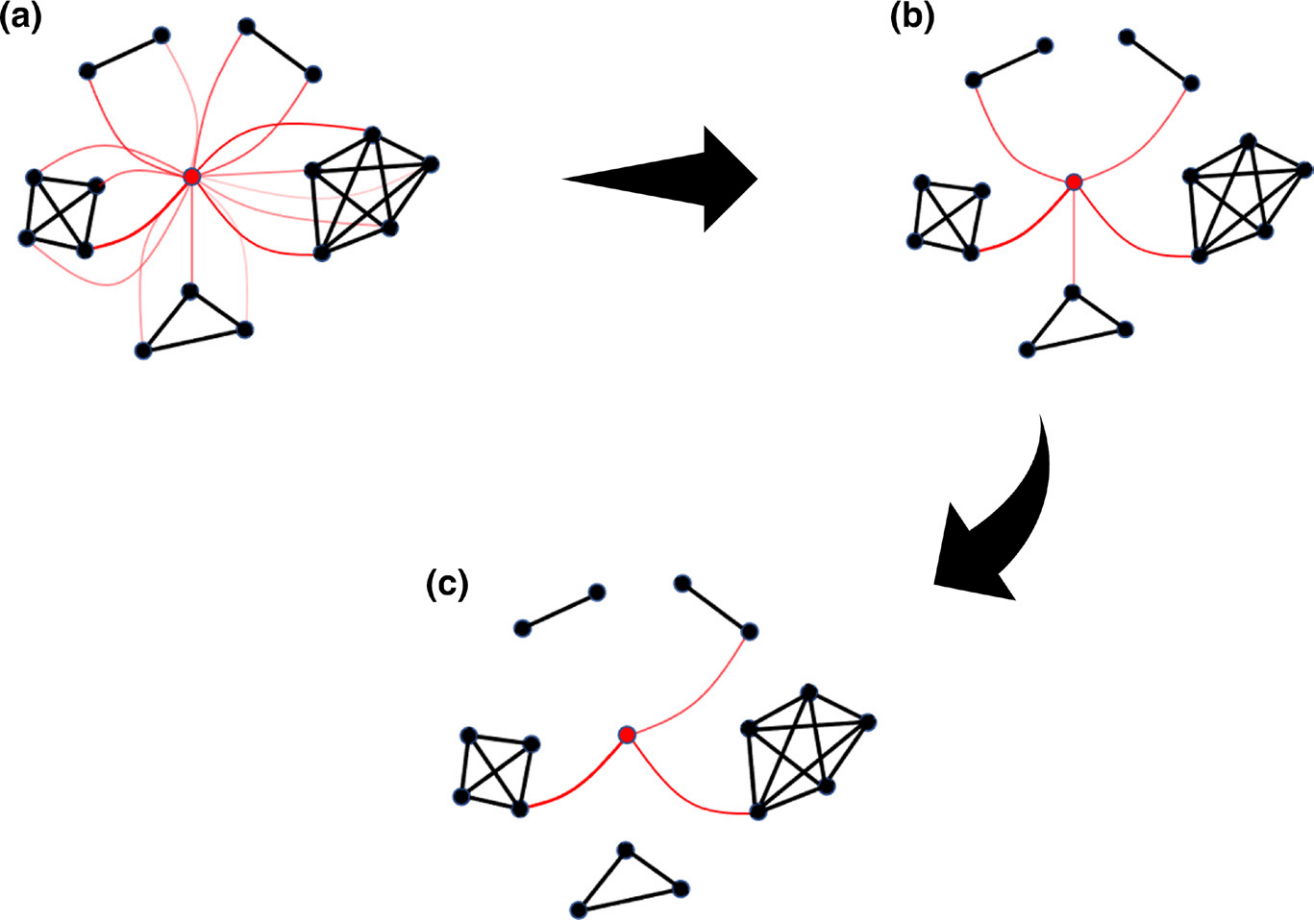
A cluster allocation of case *i* (red dot) using model (3). Cases that are in the same cluster are connected with a black line. (**a**) Red lines represent the probability of case *i* being in the same cluster with the connected case, with thicker lines indicating a greater probability. (**b**) The cluster scores of case *i*, defined by the maximum probability of case *i* being in the same cluster of case *j* over all *j* in the cluster. (**c**) The top *K* = 3 clusters for case *i* to be assigned to.

Once we have fit the model to the pairwise data and the cluster data for a collection of cases, we can use it to assign new (unsequenced) cases to clusters. Suppose that we have a new case *i* with individual data ***x***_*i*_. We compute the pairwise data ***z***_(*ij*)_ for this new case with every case in the training set (*j* = 1, …, *N*). Then we use the regression model to give a probability π_(*ij*)_, for *j* = 1, …, *N*, that the new case is in the same cluster as each of the cases in the training set; see Fig. 1(a).

Different points in the same cluster will give different probabilities of case *i* being in the cluster. To resolve these differences we assign a score to *i* for a given cluster *C* by taking the maximum of π_(_ *_ij_*_)_ over all *j* in cluster *C*: see Fig. 1(b). That is, for each cluster *C* we define

(6) $$\begin{array}{r} P_{i,C}=\max_{j\in C}(\pi_{\left(ij\right)}). \end{array}$$

We call *P*_*i,C*_ the *cluster score* of case *i* in cluster *C*. One approach is to assign case *i* to a cluster *C* if *P*_*i,C*_ exceeds a given threshold. This, however, has the effect of possibly assigning *i* to more than one cluster, or to no clusters at all. Instead, we rank the clusters according to the values of *P*_*i,C*_. We say the best K clusters for *i* are the clusters with the top K values for *P*_*i,C*_ (Fig. 1c). Continuing from the previous task, we can examine whether a new case *i* does not belong to any known clusters if for a given threshold π_0_, *P*_*i,C*_ ≤ π_0_ for all C . Furthermore, *i* creates a new cluster entirely with another unsequenced case *k* if both cases do not belong to any given clusters and π_(*i,k*)_ > π_0_.

Finally, as a benchmark, we compare the performance of our method with two other methods. The first is multinomial logistic regression, implemented on the individual variables ***x***_i_ with the cluster index as the response variable. The second is random assignment, where we ignore all variables, and select a case in the training set uniformly at random, and assign the new case to its cluster. This has the effect of assigning new cases to clusters with probability proportional to the size of the cluster.

#### Which new cases are most likely to be in a given cluster?

Though we used (6) to predict the cluster a case is most likely to be assigned to, we can turn this relationship around, and ask, for a given cluster, which unassigned cases are most likely to belong to it. This could be used, for example, to decide which of several unsequenced cases should be sequenced next, if we are interested in identifying all the cases that belong to a particular cluster.

Suppose we have parameters from training our model on a set of cases with known clusters, and let C* be a cluster of interest. We have a collection of unsequenced cases with individual data ***x***_*j*_ for *j* = 1, …, *M*, for which we have no cluster assignment. Using the method above, we compute a cluster score P_j,C*_, for *j* = 1, …, *M*. If a threshold is not chosen, we say that of the M new cases, the K cases most likely to be in cluster C* are those with the largest values of P_j,C*_,. This means that we can always pick new members for cluster C* despite their low scores. However, those cases are the best option we have to be assigned to cluster C*.

#### What is the expected cluster size after including new cases?

Again suppose that we have a cluster C* with some cases in it, and a collection of unassigned new cases. Instead of trying to determine which of the new cases belong to C*, we can estimate the total number of new cases that would be assigned to C*, and therefore estimate the true number of cases in C*. To understand the distinction from the previous task, consider a situation where there are 10 new cases that we each think are in C* with probability 0.1. Then we expect that there is going to be one new case in cluster C*, but we may not actually be able to assign any given case to C* with high probability.

Suppose we have a collection ***C*** of clusters, with cluster *C* ∈ ***C*** having $n_{C}^{0}$ initially assigned cases. Again suppose we have a collection of unsequenced cases with individual data ***x***_*j*_ for *j* = 1, …, *M*, for which we have no cluster assignment. Suppose we believe that a fraction ρ of these cases do not belong to any of the clusters in *C*. As above we compute cluster scores *P*_*j,C*_ for *j* = 1, …, *M*, and *C* ∈ ***C*** . Conditioned on a given new case *j* being in one of the clusters *C* ∈ ***C***, we propose that the probability that case *j* is in a particular cluster *C*′ is

(7) $$\begin{array}{r} q_{j,C^{\prime }}=\frac{P_{j,C^{\prime }}}{\sum_{C\in \text{C}}P_{j,C}}. \end{array}$$

Then the probability of case *j* being in cluster *C*′ is (1 – *ρ*)*q*_*j,C*_ *_’_* factoring in the fact that case *j* might not belong to any cluster. Then the expected size of cluster *C*, denoted by *n*_*C*_, is given by

(8) $$\begin{array}{r} E(n_{C})=n_{C}^{0}+(1-\rho )\sum_{j=1,\ldots ,M}q_{j,C}. \end{array}$$

### Tuberculosis data in Valencia

We apply our approach to a dataset of TB cases in Valencia, Spain, over the period between 2014 and 2016 [28]. Valencia is the fourth largest populated region in Spain, with approximately 5 million inhabitants. TB surveillance is managed by the Regional Public Health Agency (DGSP: Dirección General de Salud Pública). As a part of a mandatory routine, for each positive diagnosed TB case, clinical, microbiological and demographic data are recorded.

**Fig. 2.**
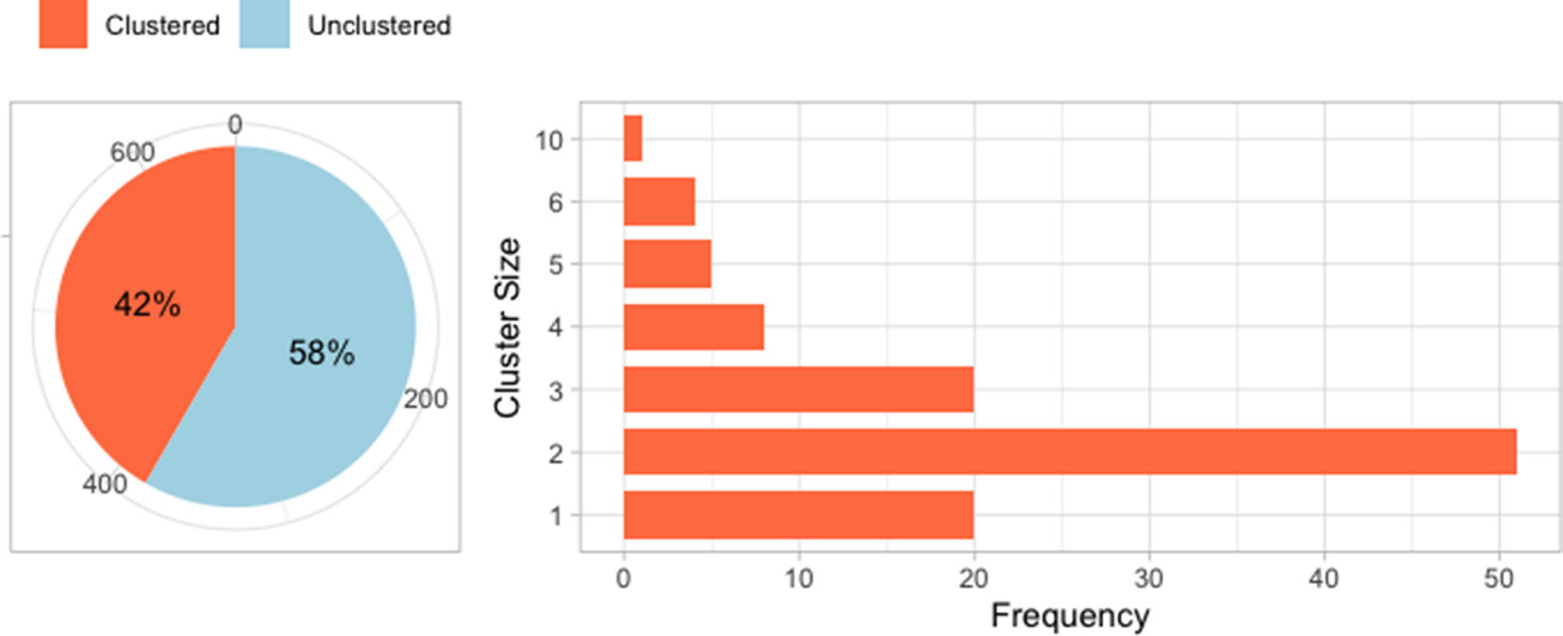
A total of 655 isolates with complete clinical/demographic data and sequences: a proportion of unclustered cases (58.34%) and cases that are classified into 109 genomic clusters (41.62 %).

A total of 785 single-patient cultures were retrieved from 18 hospitals in the Valencia region. This number corresponds to 77 % of all culture-positive cases reported by DGSP. The samples and DNA extraction method are described in a previous publication [28]. Of the 785 samples, there were 655 isolates with complete clinical and demographic data. Of these, 273 (41.62 %) cases are classified into transmission clusters using WGS, in which the clusters’ delineation is based on a cutoff of ≤ 15 SNPs. The clusters are then confirmed by building a phylogeny that includes all the isolates. This led to 109 clusters having between one and 10 cases (Fig. 2). We exclude cases in the clusters of size less than three because there is at most only one pair for each such cluster, and we cannot have some of the clusters’ cases in the training and the test set while still having at least one pair in the training set to train the model. The model can be used to predict clusters with only two cases but, in this study, having some clusters with no cases in the test set will not allow us to know the accuracy of assigning new cases into such clusters. This leaves a total of 382 unclustered cases and 151 clustered cases, which are classified into 38 clusters (Table 1).

**Table 1.** The cluster size of every cluster used in the analysis. A total of 151 cases were filtered out from 273 isolates, which results in 38 clusters

Cluster size	Cluster name
3	CL012, CL015, CL017, CL021, CL022, CL023, CL024,
	CL025, CL034, CL045, CL055, CL063, CL068, CL070,
	CL077, CL083, CL089, CL091, CL092, CL110
4	CL004, CL008, CL009, CL019, CL020, CL037, CL072
	CL078
5	CL007, CL011, CL026, CL031, CL069
6	CL001, CL003, CL010, CL016
10	CL002

We consider four categorical variables (sex, nationality, HIV status, diabetes diagnosis) and the residential location of the patient, which is recorded as latitude and longitude. Since over 10 % of Valencia’s residents are foreign-born, we could investigate, for instance, if the transmission clustering may be more associated with some nationalities than others. However, we do not use the nationality of an individual as a variable, since for most nationalities there are only a few individuals. We note that TB in the region shows a higher incidence in migrants than in local-born individuals [1]. Hence, we define nationality as having two categorical values: ‘Spanish-born’ and ‘foreign-born’.

**Fig. 3.**
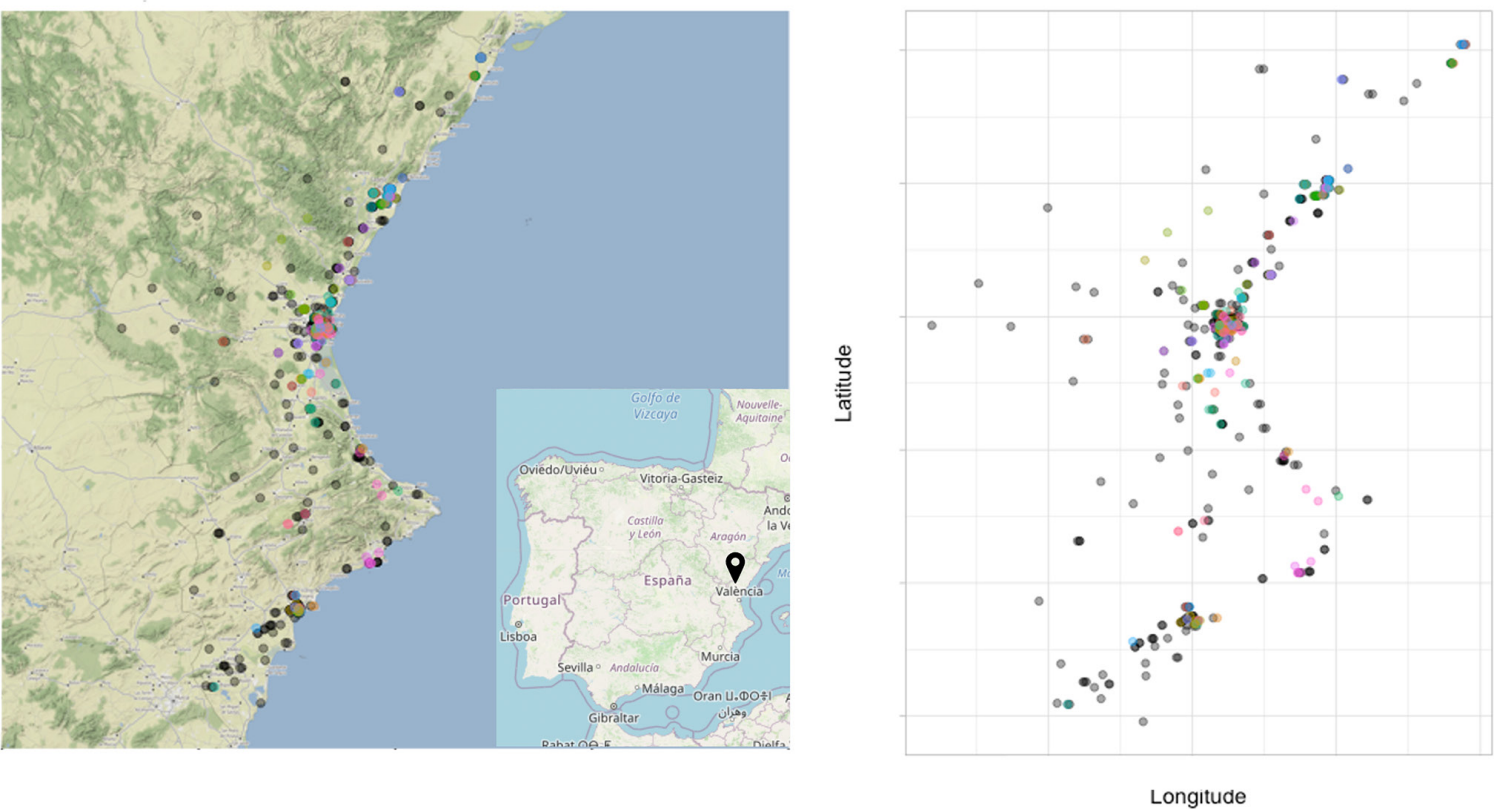
The spatial location (jittered) of the 382 unclustered cases (coloured in grey) and the 151 cases in clusters of size three or more (coloured based on their clusters) in longitude and latitude.

We consider HIV and diabetes status because both conditions are risk factors for TB: having an HIV infection increases the rate at which individuals progress from latent TB infection to active disease [29–31], and individuals with diabetes melitus have been found to have a higher rate of developing TB [32, 33]. In this study, however, we detect a low prevalence for both variables (Table 2). This may affect the estimation in our logistic regression – that is, both variables may show weak signals in the analysis, though HIV, being an infection, may correspond to contact networks that are also relevant for respiratory infections. We also consider geographical location as one of the covariates. Fig. 3 shows the jittered location of cases’ residential area and coloured by their genomic clusters.

**Table 2.** The descriptive statistics of each variable in individual data and pairwise data from the 151 isolates in clusters of size three or more. The label ‘Diff’ indicates when the two cases in a pair have different values for the categorical variable. Approximately 15 % of the region are foreign-born

Covariate	Variable	Count	
		Individual data	Pairwise data
Sex	Diff	–	4888 (43.2 %)
	Male	104 (68.9 %)	5356 (47.3 %)
	Female	47 (31.1 %)	1081 (9.5 %)
Nationality	Diff	–	4218 (37.2 %)
	Spanish-born	114 (74.5 %)	6441 (56.9 %)
	Foreign-born	37 (24.5 %)	666 (5.9 %)
Diabetes	Diff	–	2394 (21.1 %)
	Yes	18 (11.9 %)	153 (1.4 %)
	No	133 (88.1 %)	8778 (77.5 %)
HIV	Diff	–	1410 (12.5 %)
	Yes	10 (6.6 %)	45 (0.4 %)
	No	141 (93.4 %)	9870 (87.2 %)

When we compute the pairwise data for each pair of cases, each categorical variable gives two pairwise variables (Table 2). For the spatial data, we calculate the surface distance between two coordinates (latitude, longitude) using the geodist() function from the R package geodist [34] to get another pairwise variable which represents the spatial distance (in km) between two cases. This then gives us nine variables to be fitted to the pairwise logistic regression (Table 3). We also perform multinomial logistic regression, using individual variables to predict the cluster to which an individual belongs. The model performs direct categorization of individuals into one of the 38 clusters in the training data.

**Table 3.** (Top) Estimate of *β* in the pairwise logistic regression model using all nine variables. (Bottom) Estimate of *β* in the pairwise logistic regression model using only spatial distance and nationality variables

Variable	Estimation			
	$\hat{\beta }$	$s\,e\,\left(\hat{\beta }\right)$	*P*-value†	
(Intercept)	−3.392	0.254	<2e-16	***
Spatial distance	−0.018	0.002	<2e-16	***
**Sex**				
Male	0.026	0.134	0.846	
Female	0.044	0.235	0.850	
**Nationality**				
Spanish-born	0.561	0.149	1.6e-04	***
Foreign-born	1.058	0.236	7.1e-06	***
**Diabetes**				
Yes	−0.309	0.601	0.607	
No	0.079	0.154	0.606	
**HIV**				
Yes	−2.43	1.032	0.814	
No	−0.011	0.185	0.952	

**Variable**	**Estimation**			
	$\hat{\beta }$	$s\,e\,\left(\hat{\beta }\right)$	***P*** **-value**‡	
(Intercept)	−3.333	0.139	<2e-16	***
Spatial distance	−0.019	0.002	<2e-16	***
**Nationality**				
Spanish-born	0.558	0.148	1.7e-04	***
Foreign-born	1.062	0.235	6.4e-06	***

†Significance code: ***(0.0001), **(0.001), *(0.01); AIC: 2352.6

‡Significance code: ***(0.0001), **(0.001), *(0.01); AIC: 2341.4

### Model training and testing

For both pairwise logistic regression and multinomial logistic regression, to test the efficacy of our method, we split the 151 clustered cases into training and test sets with the approximate proportions 60%/40 %. However, for clusters with sizes three or four, we split the cluster such that one case is in the test set and the rest are in the training set. Thus, there are 101 cases in the training set and 50 cases in the test set. Any unclustered cases are excluded from the training set; however, they are included in the test set for the tasks described in “Do two new cases belong to the same cluster?” and “What is the expected cluster size after including new cases?”. This is because the cases’ true clusters are needed in order to measure the accuracy. Furthermore, by choosing the K clusters with the highest cluster scores we would allocate the unclustered cases to at least one of the clusters in the training set, which would underestimate the method’s performance. To test our model’s efficacy in predicting if two cases are in the same cluster and to estimate the expected cluster size (the task described in “Do two new cases belong to the same cluster?” and “What is the expected cluster size after including new cases?”), we report the performance on two different test sets, one with just 50 clustered cases and another that includes an additional 382 unclustered cases.

When choosing the test and training subsets of the data, we used the individual cases (not the pairs). Training for pairwise logistic regression was only done on those pairs where both cases were in the training set. Both training models are then tested on the test set. We repeat this procedure 1000 times in order to capture the variability due to randomly splitting the data into training sets and test sets.

We make our method available through an R package called lr2cluster, which trains the pairwise logistic regression model and allows for testing its accuracy. The package is available at https://github.com/ksusvita92/lr2cluster. The analysis scripts and figures in the study are accessible in a different repository, which can be accessed at https://github.com/ksusvita92/Genomic-Clustering.

## Results

We fit the pairwise logistic regression model to all the cases in the training set and find that three variables significantly affect the response variable (Table 3). As would be expected, greater spatial distance strongly indicates that two cases are not in the same cluster – which confirms recent results in *Mycobacterium tuberculosis* [35] – though some pairs of cases are close spatially and in different clusters. Of course, individuals may be exposed to TB in a variety of settings that are not determined by place of residence, so this is to be expected. The two other significant variables are based on nationality: if both individuals are Spanish-born, or if both are foreign-born, they are more likely to be in the same cluster. To test the correlation between spatial distance and nationality variables, we compute a correlation matrix between spatial distance data and each level of pairwise nationality data: Spanish-born, foreign-born and Spanish-foreign (or Diff). We find that the correlations are very small, ranging from −0.01 to 0.01 (Fig. S1). We also show a summary table of the regression coefficient, *β*, when we fit the model using only spatial distance and nationality variables in Table 3. Note, however, that coefficient and significance estimates are impacted by dependence in the data: if cases *A* and *B* are in the same cluster, and *B* and *C* are in the same cluster, then necessarily, *A* and *C* are in the same cluster. The response variable is therefore not independent across pairs. Recent work develops statistical models to account for this [36], but here, our focus is on the predictions and their applications.

**Fig. 4.**
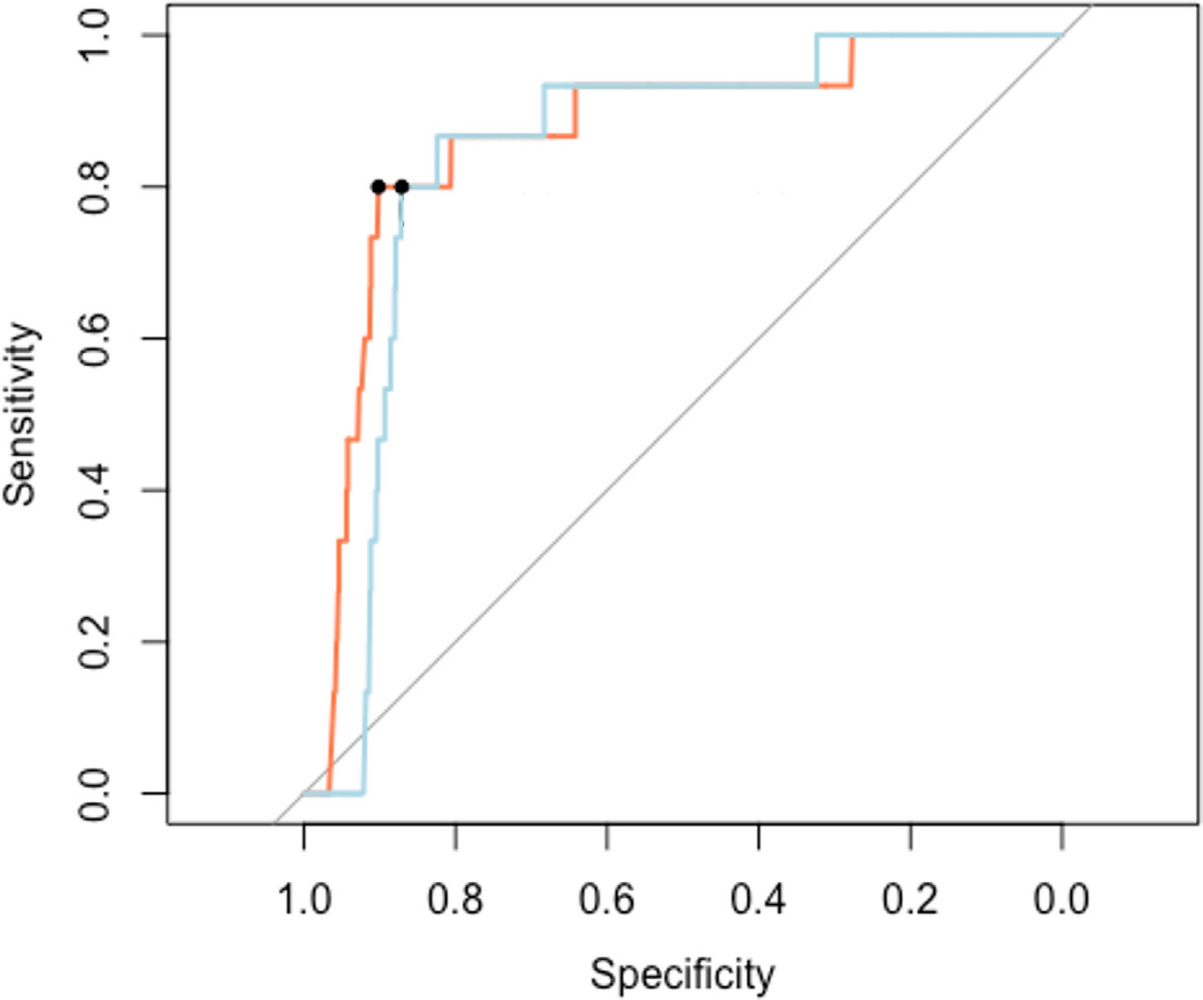
ROC plots from cases in the test set excluding the unclustered cases (orange line) and including the unclustered cases (light blue). The black dots represent the specificity and the sensitivity of our model evaluated at the optimum threshold $\pi_{0}$ .

We have four applications of the model. First, we use our method to predict whether a pair of unsequenced cases are in the same cluster, as described in “Do two new cases belong to the same cluster?”. We vary the decision threshold π_0_ from 0 to 1 to determine the receiver operating characteristic (ROC) curve, and also estimate an optimal π_0_ using (5) given a cost ratio *c =* 13 for the test set excluding the unclustered cases and *c =* 1002 for the test sets including the unclustered cases. Fig. 4 shows the ROC curves of one sample of our trained model fitted on the pairwise data from the cases in the test set when we include and exclude unclustered cases. We obtain the same threshold π_0_ = 0.040 with 90.17 % specificity, 80.00 % sensitivity and 90.04 % accuracy for the test set excluding the unclustered cases, and 87.09 % specificity, 80.00 % sensitivity and 87.09 % accuracy for the test set including the unclustered cases.

**Fig. 5.**
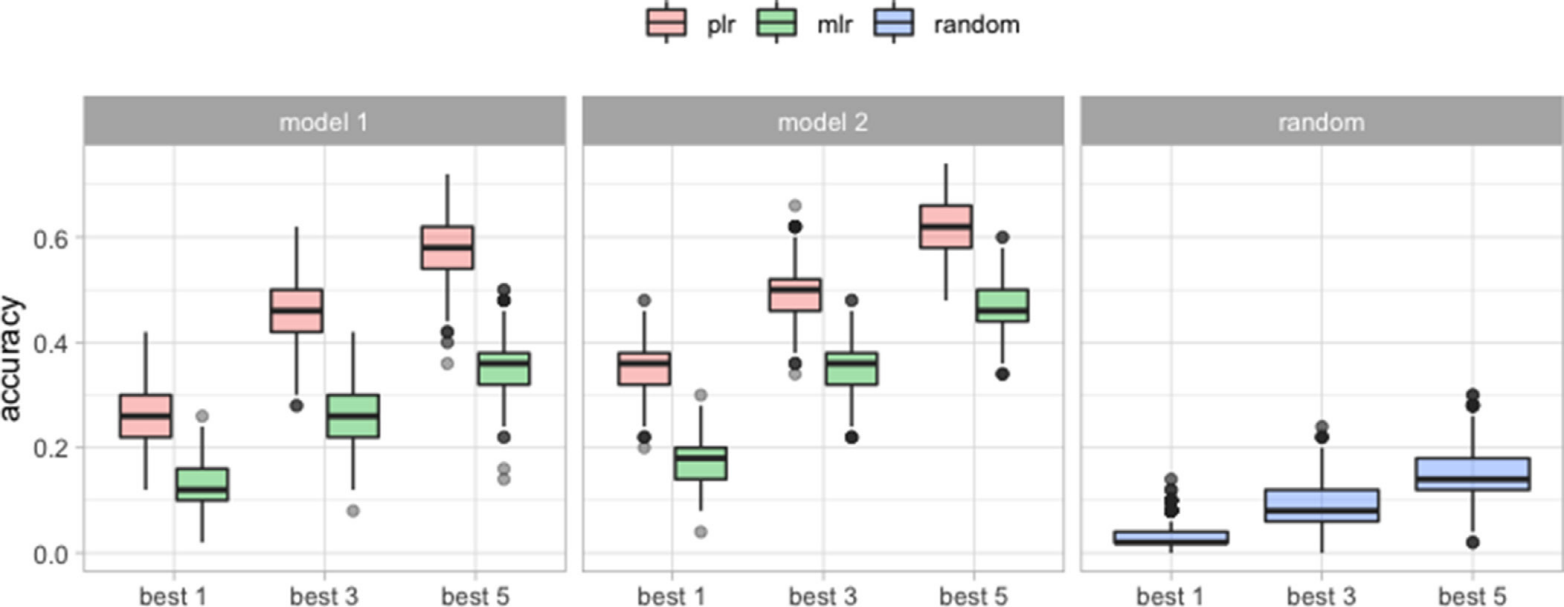
The accuracy of predicting the correct cluster among the top *K* = 1, 3, 5 clusters provided by the model, over 1000 trials. Model 1 is fitted on all the variables. Model 2 is fitted only on spatial location and nationality.

The second application is to predict the *K* most likely clusters for each case in the test set, as described in “Which cluster is an unsequenced case most likely to belong to?”, for *K* = 1, 3, 5. We split the data into a training set and a test set 1000 times. For each trial, we train the model and do cluster assignments on cases in the test sets and then compare our results with their true clusters. The accuracy for a given *K* is the fraction of new cases whose true cluster is in the best K clusters predicted by our model. For example, suppose the true cluster of case i is *C* ^∗^. If *P*_*i,C^∗^*_ is ranked as the second highest, we say that the pairwise logistic regression correctly classifies i when *K* = 3, 5, but not when *K* = 1. To compute the accuracy of the best *K* clusters by the other methods, we pick the clusters with the *K* highest predicted probabilities (for the multinomial logistic regression), and pick *K* clusters randomly with probabilities proportional to the clusters’ size (for the random assignment). Table 4 shows the mean accuracy and its standard deviation over 1000 trials for pairwise logistic regression, multinomial logistic regression and random assignment. We also compute the accuracy when we fit both pairwise logistic regression and multinomial logistic regression using all of the available variables, and only the significant variables: spatial distance and nationality. Both logistic regression models do better than random assignment (Table 4 and Fig. 5). Furthermore, pairwise logistic regression has higher mean accuracy than the multinomial logistic regression for predicting the best *K* clusters. Using only significant variables for both logistic regression models also increases the average accuracy substantially.

**Table 4.** The mean accuracy over 1000 repetitions of pairwise logistic regression, multinomial logistic regression, and random assignment. The logistic regression models are fitted using two sets of variables: {spatial distance, nationality, sex, HIV, diabetes} and {spatial distance, nationality}

	1-best		3-best		5-best	
	Mean	sd	Mean	sd	Mean	sd
Model 1*						
Pairwise LR	0.261	0.048	0.454	0.059	0.578	0.056
Multinom. LR	0.129	0.036	0.259	0.048	0.351	0.054
**Model 2**†						
Pairwise LR	0.352	0.043	0.496	0.050	0.619	0.046
Multinom. LR	0.173	0.038	0.357	0.047	0.467	0.049
Random	0.029	0.024	0.088	0.040	0.147	0.049

*Fitted model with spatial location, nationality, sex, HIV and diabetes.

†Fitted model with spatial location and nationality.

**Fig. 6.**
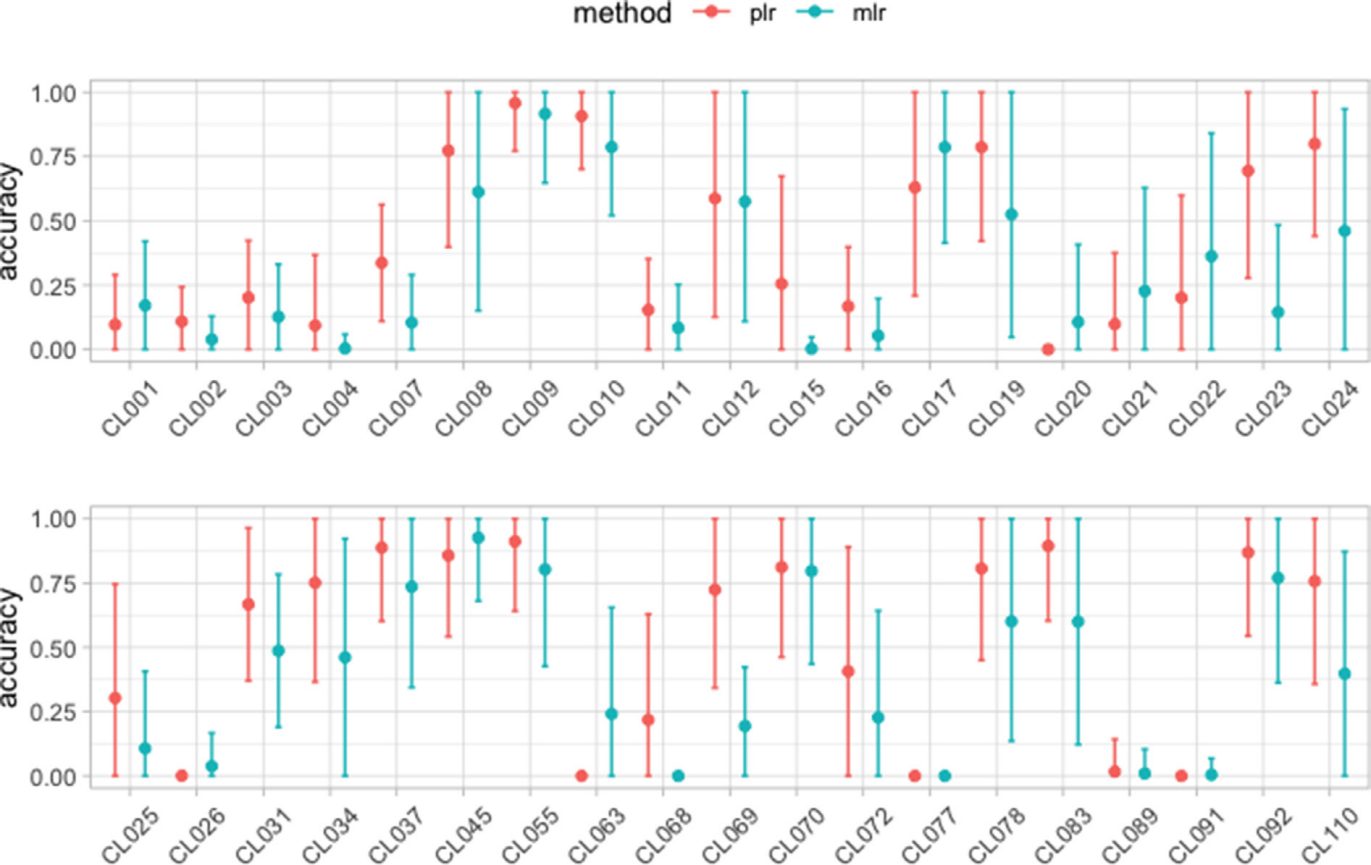
The average fraction of each cluster’s predicted ‘most likely’ new cases that were actually in the cluster, along with standard error, over 1000 repetitions. For each cluster in the training set, these were the five cases in the test set that were most likely to be in the cluster. Dots represent the mean accuracy over 1000 repetitions of the test/train split, and the error bars show one standard error from its mean.

Using the same trained model from the first and second applications, we also determine which of the cases in the test set are most likely to belong to each of the clusters in the training set, as described in “Which cluster is an unsequenced case most likely to belong to?”. To compare how well the pairwise logistic regression and the multinomial logistic regression perform, for each cluster we select five unsequenced cases with the highest match to that cluster. The accuracy in this context is the fraction of these five that are indeed in this cluster. Fig. 6 shows the accuracy for both models (pairwise and multinomial logistic regression) in each cluster. Typically, but not always, the pairwise logistic regression performs better than the multinomial logistic regression.

**Fig. 7.**
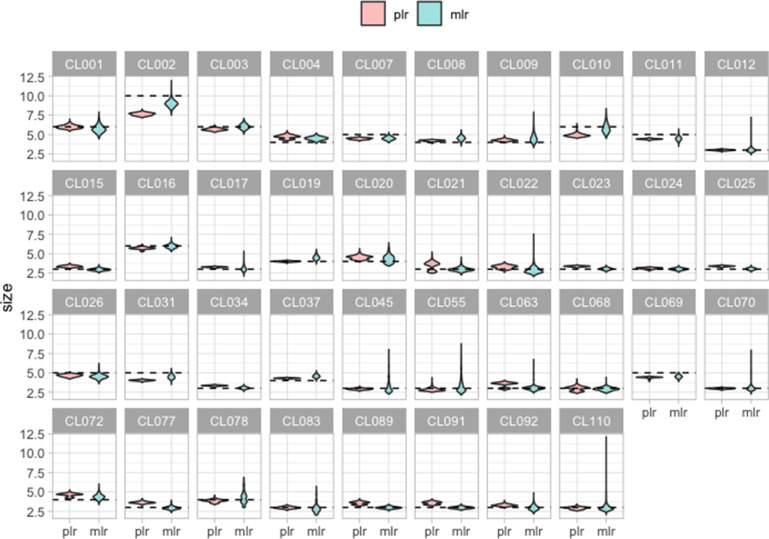
The expected cluster size, predicted using multinomial logistic regression and pairwise logistic regression over 1000 repetitions, excluding unclustered cases. The dashed line represents the true cluster size.

The final application is to estimate the expected cluster size if all cases in the test set were correctly identified in their correct cluster, as described in “What is the expected cluster size after including new cases?”. We use (8), where for any cluster *C*, $n_{C}^{0}$ is the number of cases in the training set that belong to *C* and *ρ* is the proportion of unclustered cases in the test set. We set *ρ* to be the true fraction of unclustered cases in the test set, though in practice this would have to be estimated. As described above, we use two test sets, one with unclustered cases excluded (and hence *ρ = 0*) and one with 382 unclustered cases (and hence *ρ* = 88.43%). We found that the estimates of the expected cluster size are identical when we exclude or include the unclustered cases. This is because, although our method forces the unclustered cases to be assigned to the given clusters, their cluster scores are very low and so they do not impact the expected sizes. In Fig. 7, we present the expected cluster size using both pairwise and multinomial logistic regressions. The results are similar for both regression models, though pairwise logistic regression has lower variability. Several clusters (CL021 and CL063 for example) have multi-modal predicted cluster sizes (Fig. S2). Some clusters contain a small number of cases that are quite spatially distant from the rest of the cluster. Whether these happen to land in the training data can impact the statistical model, resulting in two sets of predicted sizes, and accounting for the downward bias in some cluster size predictions (Fig. S3).

## Discussion

We have developed a method to assign newly identified cases of an infectious disease to existing transmission clusters using several data streams. Starting with a training set with pre-defined (known) clustering, we train a logistic regression model to map pairwise similarity data to a binary variable that indicates whether two cases are in the same cluster. We use the model for several applications: determining whether two unsequenced cases are likely to cluster together, deciding which cluster an unsequenced case of interest is most likely to be in, deciding which unsequenced cases are most likely to be in a cluster of interest, and estimating the true sizes of known clusters given a set of unsequenced cases. These applications may be useful to monitor which clusters should be prioritized for interventions [37–41].

The coefficients of the trained model can help to indicate which variables are important for determining clustering. We implement our method on TB incidence data in Valencia, Spain, between 2014 and 2016. The data are relatively small, and thus we would like to emphasize that the results in this work represent a special instance of the prediction of TB clustering in general, and may not generalize to outbreaks in other settings or of other diseases. In our analysis, we train a logistic regression model on a subset of data to determine which cluster a case is allocated to, and then compare the findings with the clusters identified by WGS data. To verify if some metadata have significant signals in identifying the current clusters, we perform statistical testing on each regression coefficient of the pairwise logistic regression, which is shown in Table 3. The method will omit the variables if their *P*-value is more than 5%, and conclude that those covariates are less important. For example, HIV and diabetes show weak signals in this analysis, though this result may differ in other settings. We found that being close spatially and having the same nationality status (Spanish-born or foreign-born) was associated with an increased likelihood of being in the same cluster.

We compared our method to multinomial logistic regression, which tries to directly predict clusters from the new case’s individual data, and we compared to a random assignment of the new case to existing clusters. Although the accuracy is not very high in absolute terms, our method outperforms both approaches in predicting the best *K* clusters, for *K* = 1, 3, 5, as seen in Fig. 5 and Table 4. Our method performs well in estimating the expected cluster size. Although multinomial logistic regression performs equally well, the pairwise logistic regression has lower variance. When we consider whether two unsequenced cases are in the same cluster, the pairwise logistic regression model performs well with an area under the curve (AUC) of 86.55 % for the test set excluding the unclustered cases and 84.17 % for the test set including the unclustered cases. A multinomial regression cannot be used for this application, because it can only ever predict whether cases are in the particular set of known clusters on which it was trained. In contrast, our approach can be used to detect previously unknown clusters among unsequenced cases, by predicting whether the unsequenced cases cluster together even if they would not group with known clusters.

Our work was in part inspired by Cori *et al.* [23] who used multiple data sources (or data streams) to group cases into clusters. For each data stream (time, spatial location, genetic distance, etc.), a graph was created in which two cases were linked if they were closer than a defined threshold for that data stream. If an edge existed between the cases in the graph for each data source, then an edge was placed between the cases in a final graph, and clusters were connected components of this graph. For example, two cases might be linked if their pathogens had very similar sequences (linked in the genomic data graph), they occurred at nearby points in time (linked in the ‘time’ graph) and they were at nearby spatial locations (linked in the spatial graph). Naturally this process depends on the thresholds used for each data stream. As more data streams are added, the clustering conditions either become more stringent, or the thresholds need to be adjusted to compensate. An advantage of their approach is that no cluster information is needed for training.

Our model has several advantages. Instead of inferring clusters from knowledge of the disease in question through the selection of thresholds for each variable [23], our method starts with a dataset that already has information about clusters, and then infers coefficients in a logistic regression model. The coefficients in the model are readily interpreted as the contributions that variables make to the odds ratio that two cases are in the same cluster. If a particular covariate is not relevant for clustering, for example HIV status or diabetes diagnosis in our data, its impact on the response variable will be small and it will be deemed non-significant, which suggests omitting it from subsequent models. Furthermore, since logistic regression does not involve using hard thresholds, that two cases are distant according to a particular variable is not enough to say they are not in the same cluster; agreement on several variables with large coefficients is enough to give a high score even when they disagree on other variables. This seems suitable for a chronic infection such as TB where the location and timing of case detection will be somewhat informative, but not at all definitive, about the location and timing of infection and consequently the clustering. We anticipate that our approach is more transportable from one setting to another than an approach based on predicting the cluster membership directly, as in multinomial logistic regression. A multinomial logistic regression model whose response variable is the membership in the clusters obtained in Valencia, Spain, for example, cannot be used to predict cluster membership for TB cases in another location, but a pairwise regression model could be used, even without training specific to the new setting, in a new setting. This is fundamentally because the differences between cases in the new setting – in space, genomic distance and so on – can be comparable to (for example) Valencia even when the latitude and longitude, the time, or the genomic sequence of *individual* cases are far from the locations, times and sequences in Valencia. Although the prediction is based on the model trained in the original setting, if the new setting has the same variables (in this particular case, distance and nationality variables) we could use the model in the new setting to make predictions, albeit with uncertain, and lower, accuracy. When new data are available, the model can be retrained, potentially with new variables. However, the original model provides a good starting point in the absence of new data. In this way, a pairwise model is likely to be more generalizable than a model predicting the clusters directly.

Our approach has a number of important limitations. A minor one is that we have assumed that the reported cases are well documented. While cases with partially missing data are discarded in the current analysis, imputation could be used prior to training our model and/or prior to using the model for prediction. Our dataset is relatively small, and for some small clusters, whether individual cases are included in the training set or not causes substantial differences in the predictions. This would probably be improved with a larger dataset, though even there, if there are small clusters (of size 3–10, for example), the effect would remain. More fundamentally, in many of our analyses, we include unclustered cases (except for computing the accuracy of the task described in 'Which cluster is an unsequenced case most likely to belong to?' and 'Which new cases are most likely to be in a given cluster?'). This means that our results apply to unsequenced cases, conditional on those cases being very likely to be clustered. The connection to the logistic regression framework in principle allows comparison of the relative significance of the variables to the clustering. However, as mentioned above, the response variables are not independent, and this is likely to cause the model to make optimistic estimates of the statistical significance of the estimated coefficients *β* [36]. However, this should not greatly impact the quality of the cluster predictions.

A more fundamental limitation lies in the notion of clustering as the basis for prediction at all. First, whether cases lie in clusters, and with which other cases, will depend on the clustering method used and its parameters [42]. Next, whether clusters are a good description of the population structure in the data will depend on the sampling and on the epidemiology; for an obligate human pathogen, if we truly were to sample every infection, each case would necessarily be linked to another. Cluster structure in genomic data arises due to the fact that we typically do not have samples from all infections, from other jurisdictions or from environmental reservoirs in some pathogens. Finally, there are several ways that two cases can be in the same cluster: they may be directly linked, or they may be linked only through some large or small number of intermediates. These issues are relevant in any analysis centring on clustering.

Despite these limitations, clustering remains a fundamental tool in genomic epidemiology, and clusters are often the basis of reporting and onward analysis [43]. We envisage that an application of our work will be to inform strategies for genomic surveillance, directing sequencing towards cases likely to be in particular clusters of interest for public health, and determining the resources and sequencing volumes likely to be needed to achieve stated surveillance goals, given a context in which sufficient isolates have already been sequenced. This process could be iterative, allowing ongoing definition and investigation of high-priority clusters, and allowing results from intensive sequencing studies to be integrated with ongoing routine genomic surveillance. We hope the simplicity and the flexibility of our method will make it an effective and practical tool to aid infectious disease surveillance and genomic epidemiology.

## Supplementary Data

Supplementary material 1
