# Supplementary material for: Epidemiological cluster identification using multiple data sources: an approach using logistic regression
Source: Microb Genom. 2023 Mar 3;9(3):mgen000929. doi: 10.1099/mgen.0.000929 (PMC10132077; doi:10.1099/mgen.0.000929)
Supplement: Supplementary material 1 [file mgen-9-929-s001.pdf]

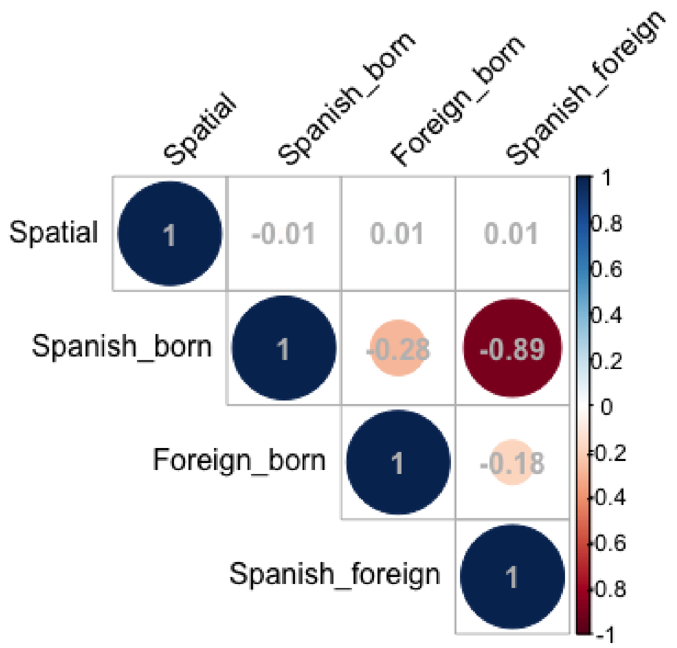

Fig. S1. The correlation plot between spatial distance variable and nationality. The pairwise transformation of nationality data results in a categorical variable with 3 levels: Spanish-born, foreign-born, and Spanish-foreign (or Diff). The correlation is computed between spatial distance data and each level of nationality data.

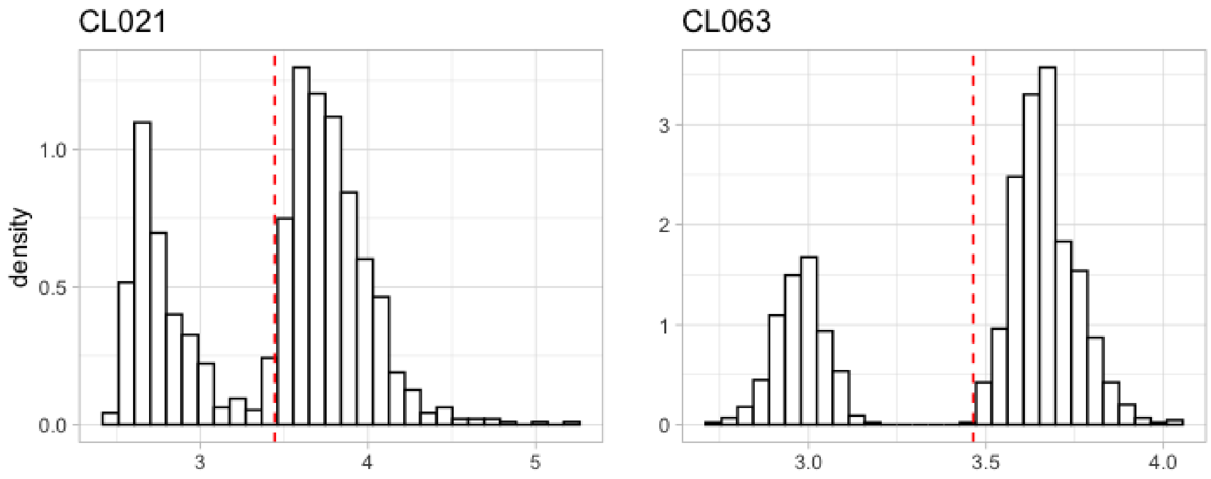

Fig. S2. Expected cluster size distribution of cluster CL021 and CL063. The red dashed lines represent the true size of each cluster. The bimodal distributions for CL021 and CL063 arise because some choices of the test-train split omit a spatially-distant case from the training set, leading the model to estimate that close spatial proximity is required for membership in CL021 and CL063.

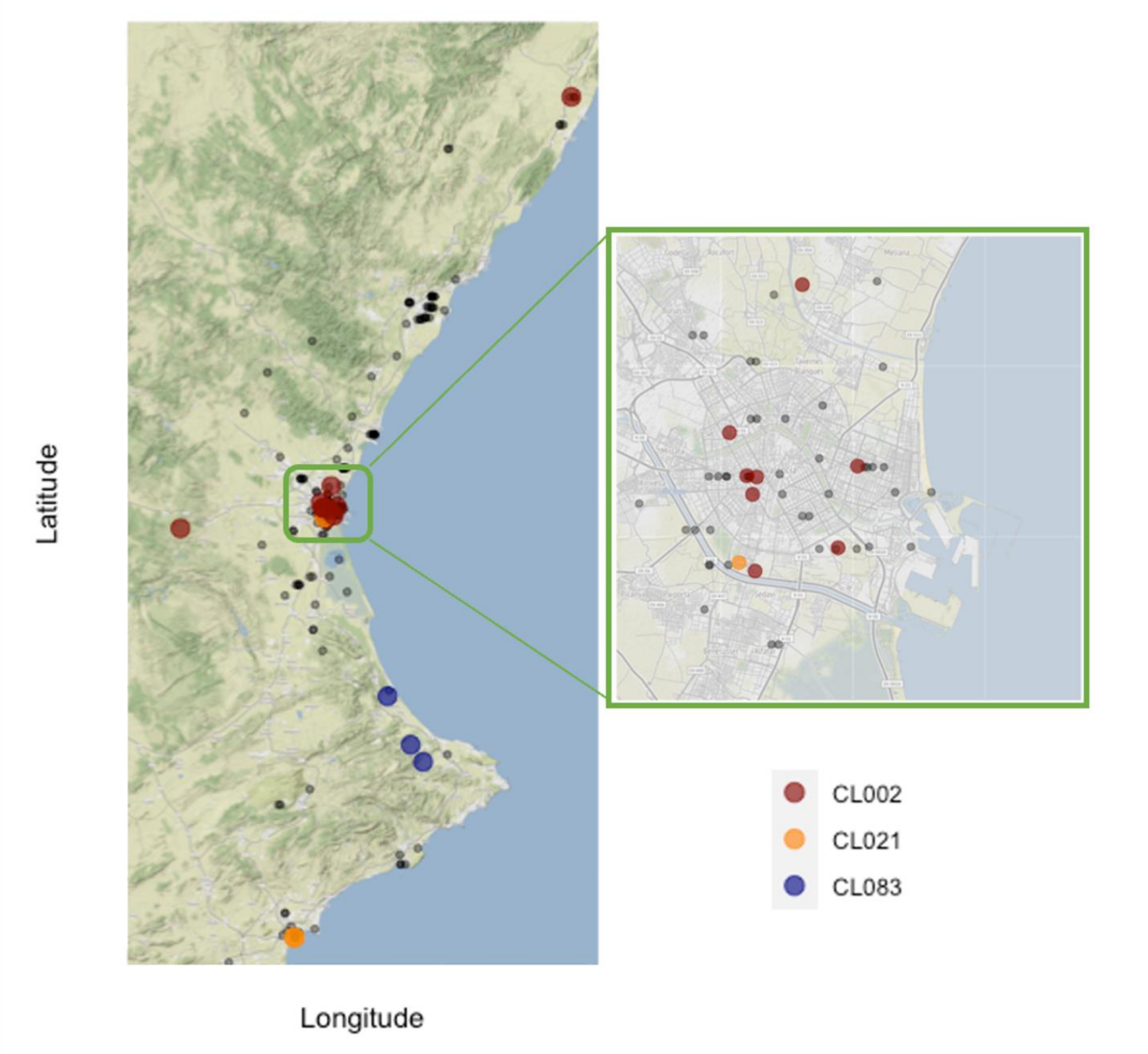

Fig. S3. The spatial location of cases in three of the clusters are highlighted (Clusters CL002, CL021, and CL083); grey dots are cases from other clusters. Cluster CL002 has two members that are spatially distant from the majority of the cases. Many choices of the test/train split will place both of these in the test set simply by chance, and the resulting statistical model will be built only on the (lower) spatial distances among the pairs located within Valencia.
